# Supplementary material for: An open repository of real-time COVID-19 indicators
Source: Proc Natl Acad Sci U S A. 2021 Dec 13;118(51):e2111452118. doi: 10.1073/pnas.2111452118 (PMC8713778; doi:10.1073/pnas.2111452118)
Supplement: Supplementary File [file pnas.2111452118.sapp.pdf]

PNAS
www.pnas.org

Supplementary Information for

An Open Repository of Real-Time COVID-19 Indicators

Alex Reinhart, Logan Brooks, Maria Jahja, Aaron Rumack, Jingjing Tang, et al.

Alex Reinhart
E-mail: areinhar@stat.cmu.edu

This PDF file includes:

Figs. S1 to S13 (not allowed for Brief Reports)

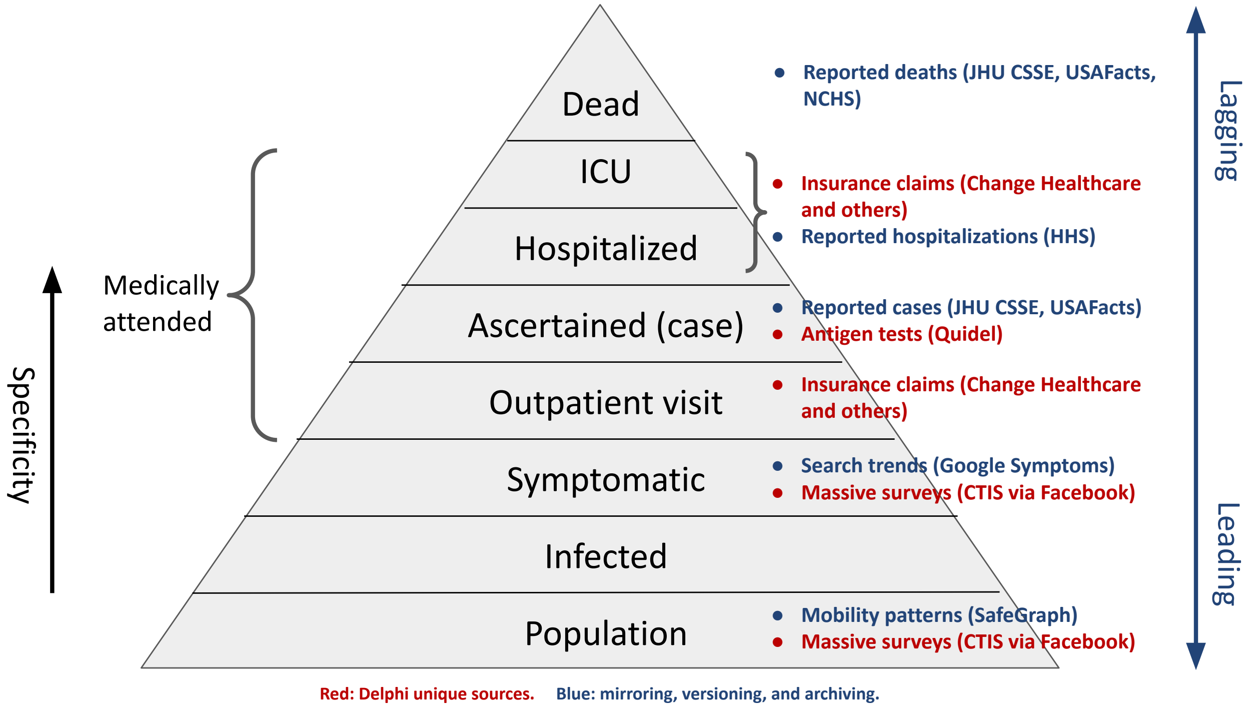

Fig. S1. Epidemiological "severity pyramid", representing the progression of disease progression, from relevant public behaviors, through infection, towards increasingly severe stages of disease. The annotations here refer to the data sources available in Delphi's Epidata API.

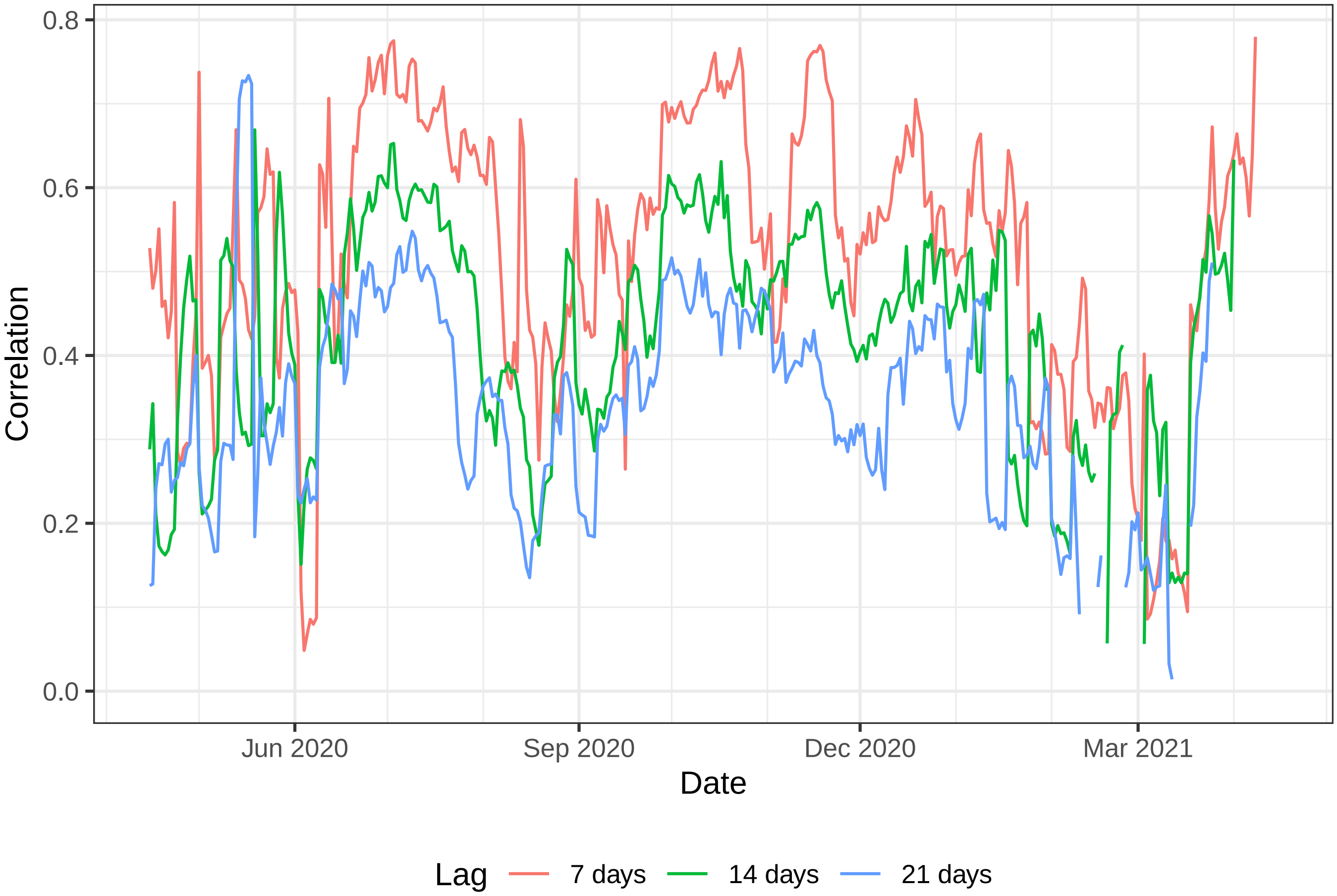

Fig. S2. Geo-wise correlations between cases and lagged cases 1, 2, or 3 weeks prior, for all counties in the U.S. Lagged cases are correlated with cases as one might expect, but note the precipitous drop in correlation in February 2021. This matches the correlation drop between other COVIDcast signals and cases during the same time period, supporting the hypothesis that the drop was due to decreased heterogeneity in case rates by county.

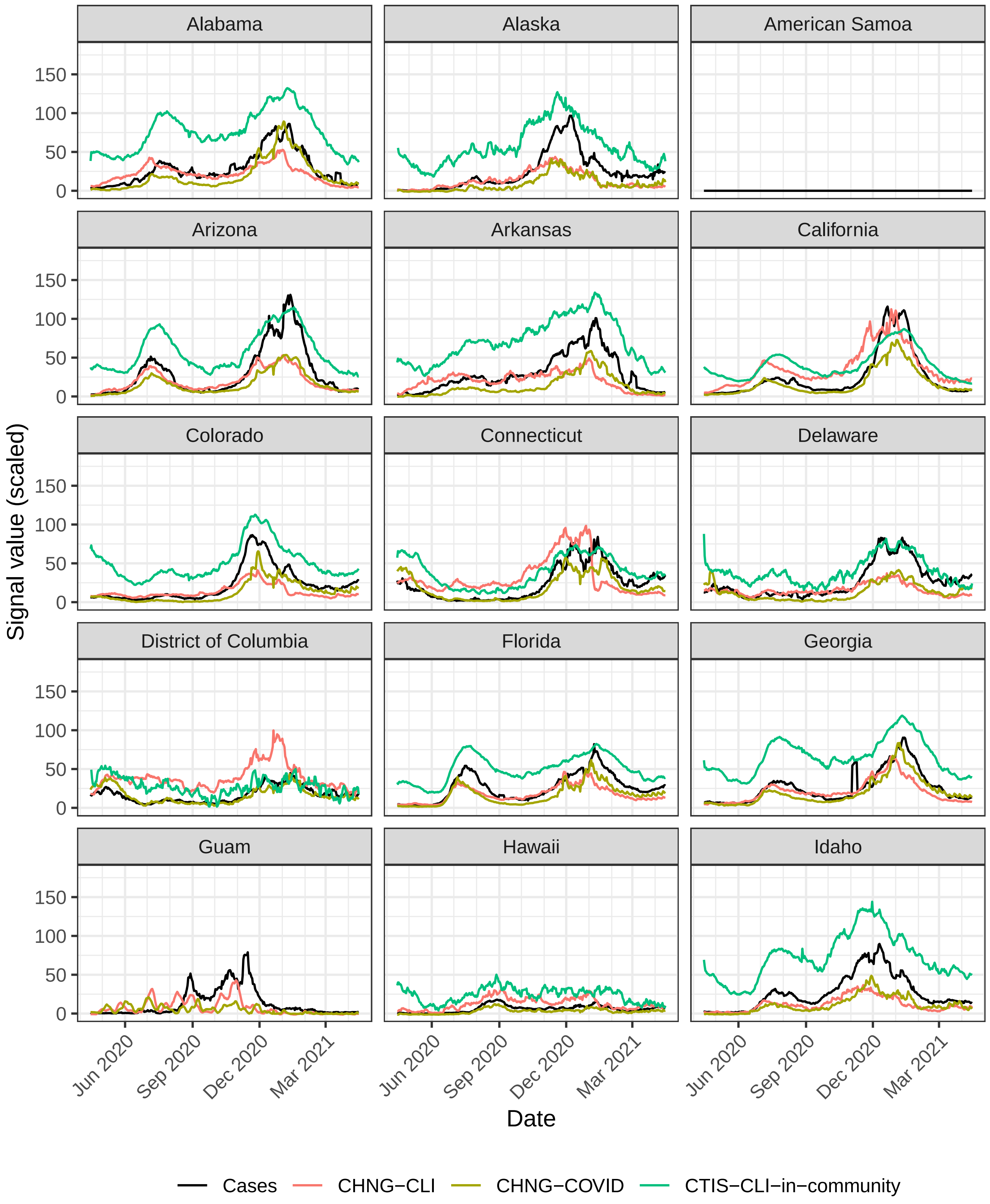

Fig. S3. Trends of cases, CHNG-CLI, CHNG-COVID, and CTIS-CLI-in-community for U.S. states and territories. Cases are displayed on the rate scale: counts per 100,000 people. Other signals are scaled to have the same global range across all counties and times. (Part 1 of 4.)

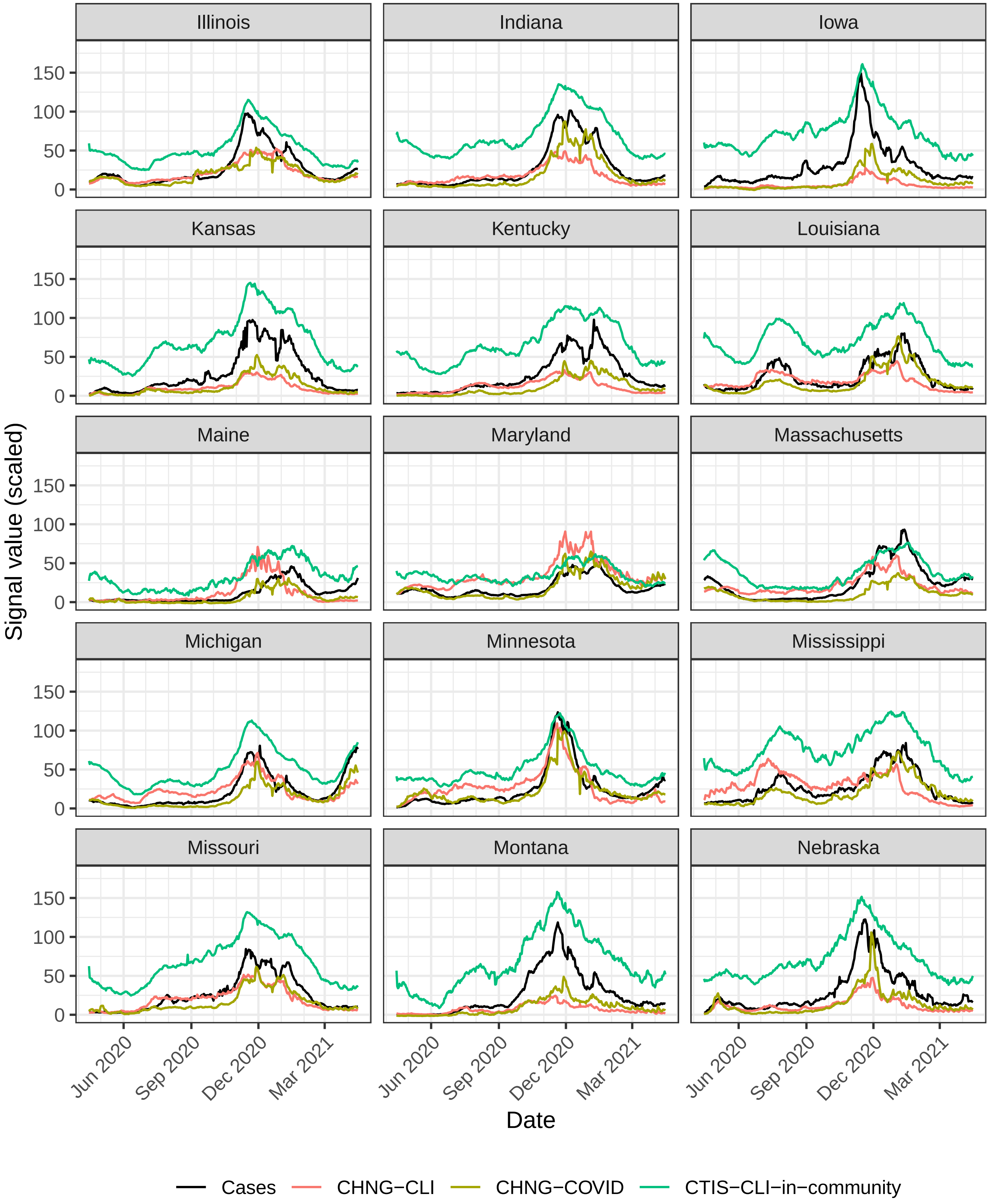

Fig. S4. Trends of cases, CHNG-CLI, CHNG-COVID, and CTIS-CLI-in-community for U.S. states and territories. (Part 2 of 4.)

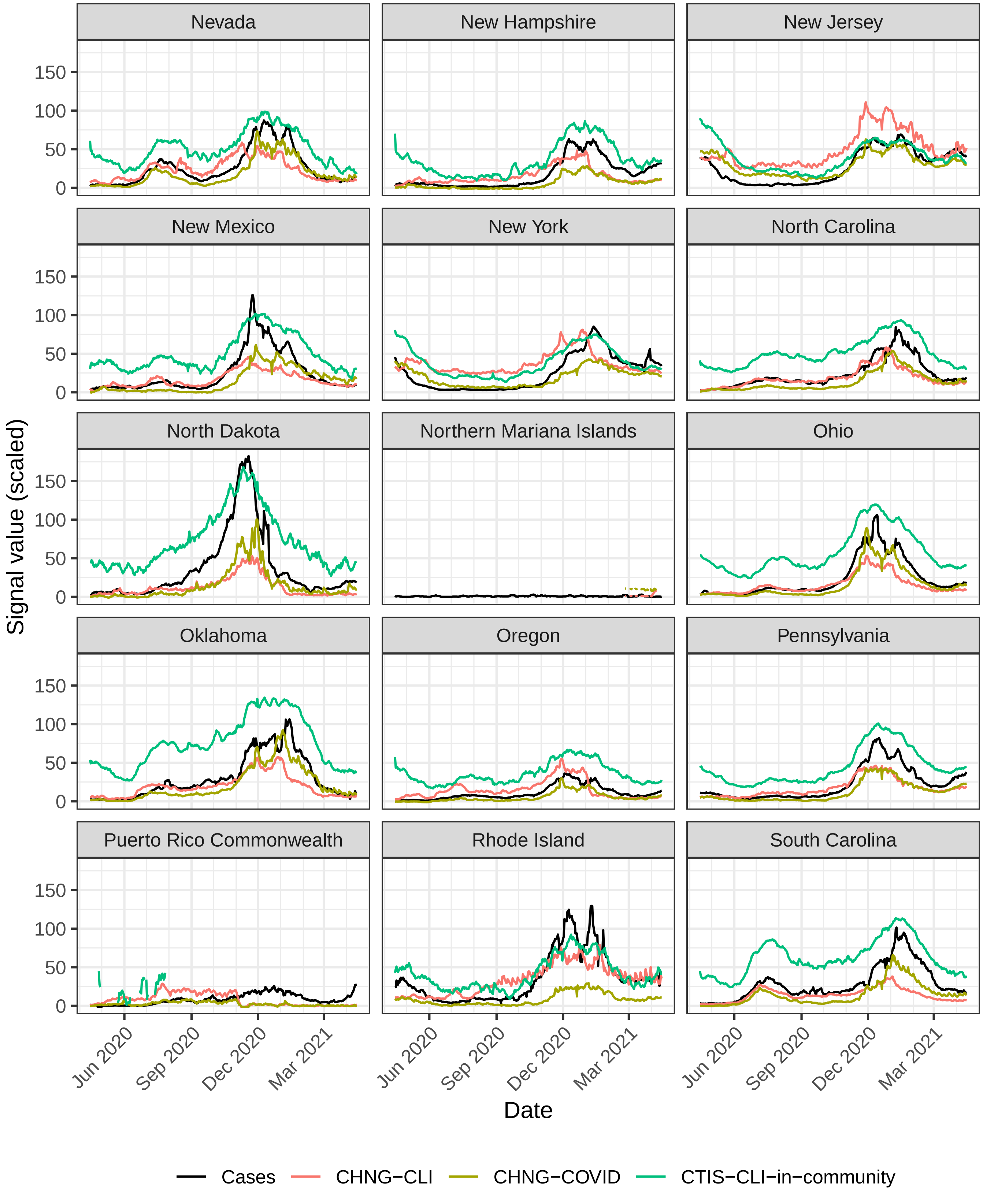

Fig. S5. Trends of cases, CHNG-CLI, CHNG-COVID, and CTIS-CLI-in-community for U.S. states and territories. (Part 3 of 4.)

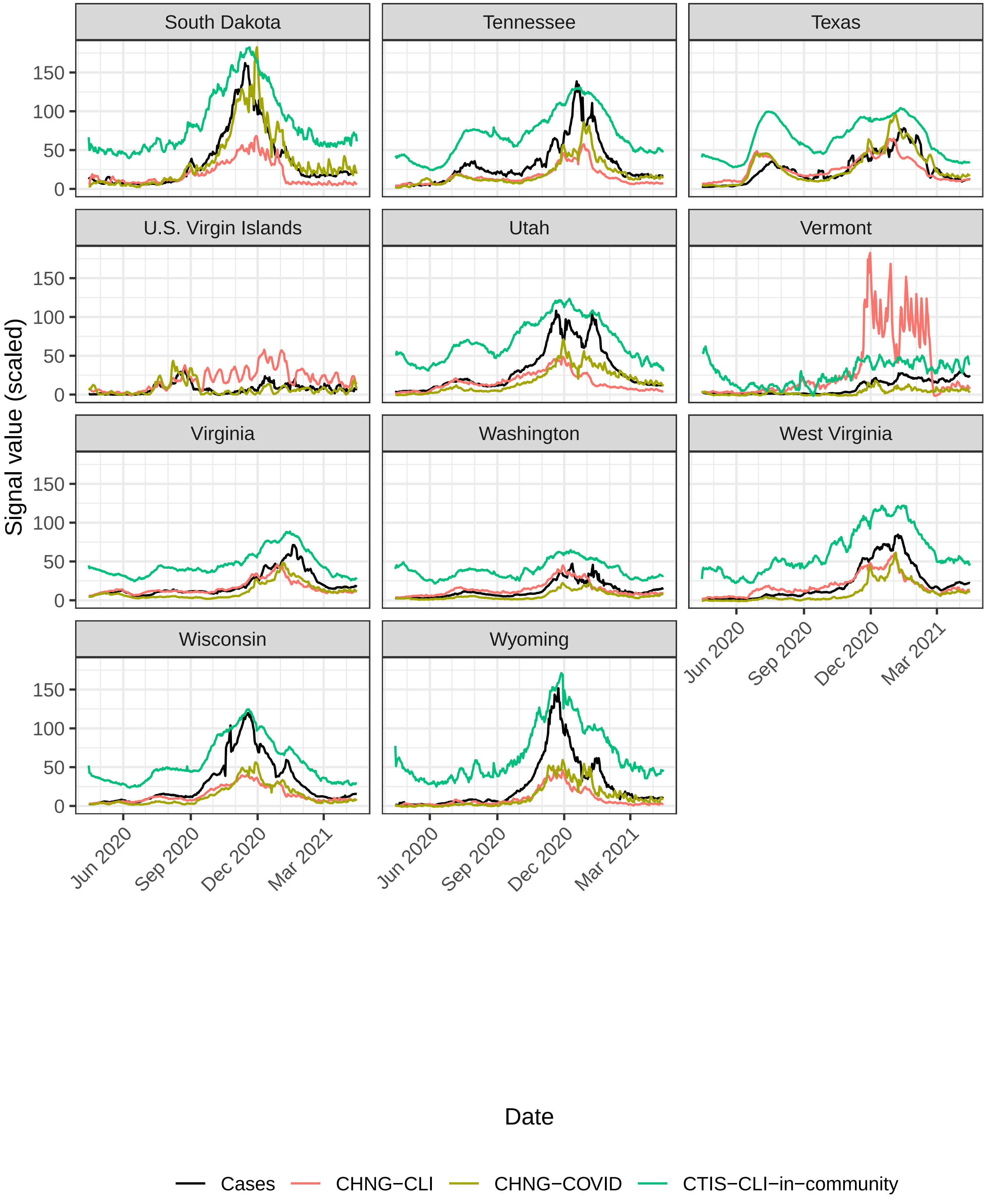

Fig. S6. Trends of cases, CHNG-CLI, CHNG-COVID, and CTIS-CLI-in-community for U.S. states and territories. (Part 4 of 4.)

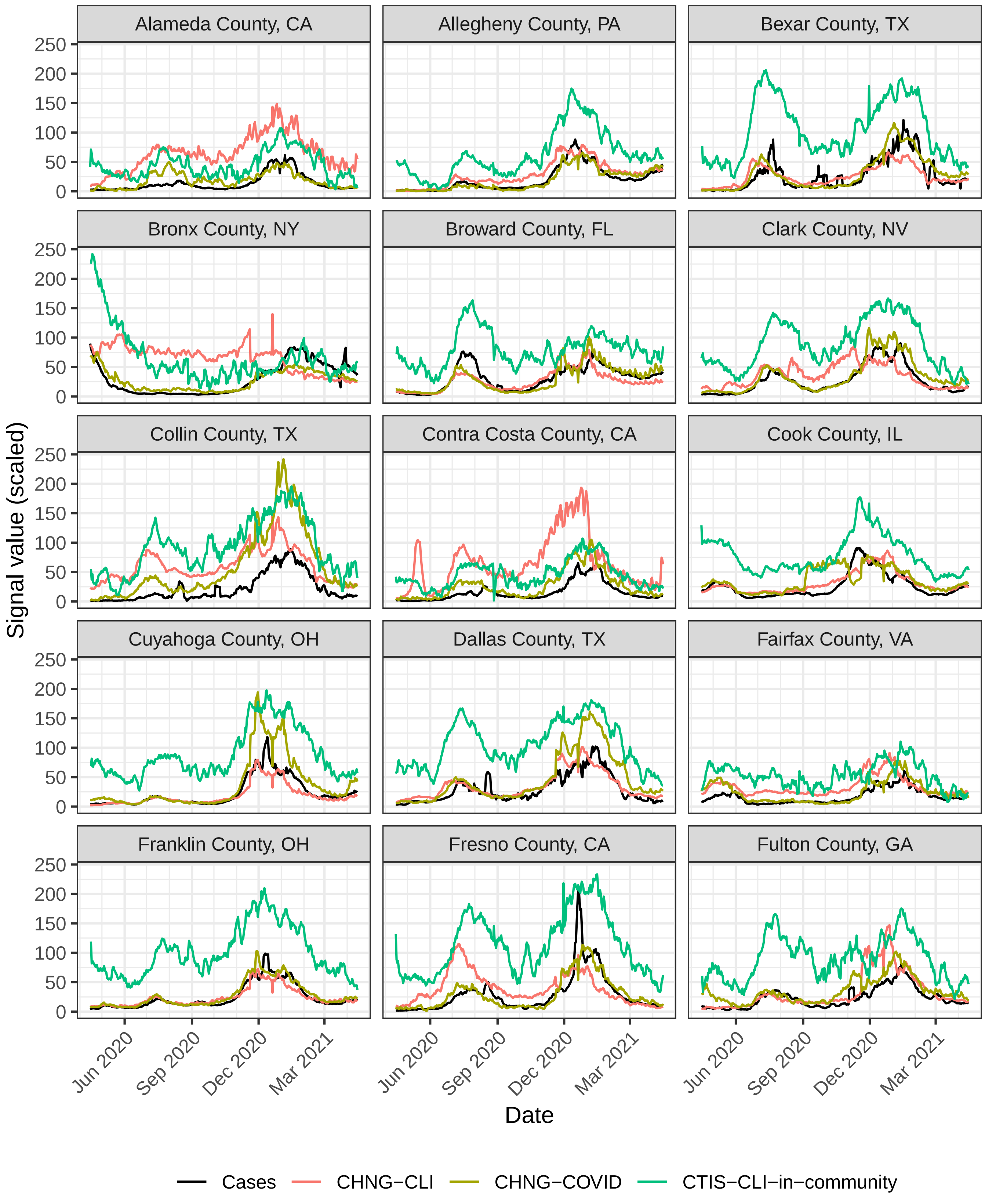

Fig. S7. Trends of cases, CHNG-CLI, CHNG-COVID, and CTIS-CLI-in-community for the 50 most populous U.S. counties. Cases are displayed on the rate scale: counts per 100,000 people. Other signals are scaled to have the same global range across all counties and times. (Part 1 of 4.)

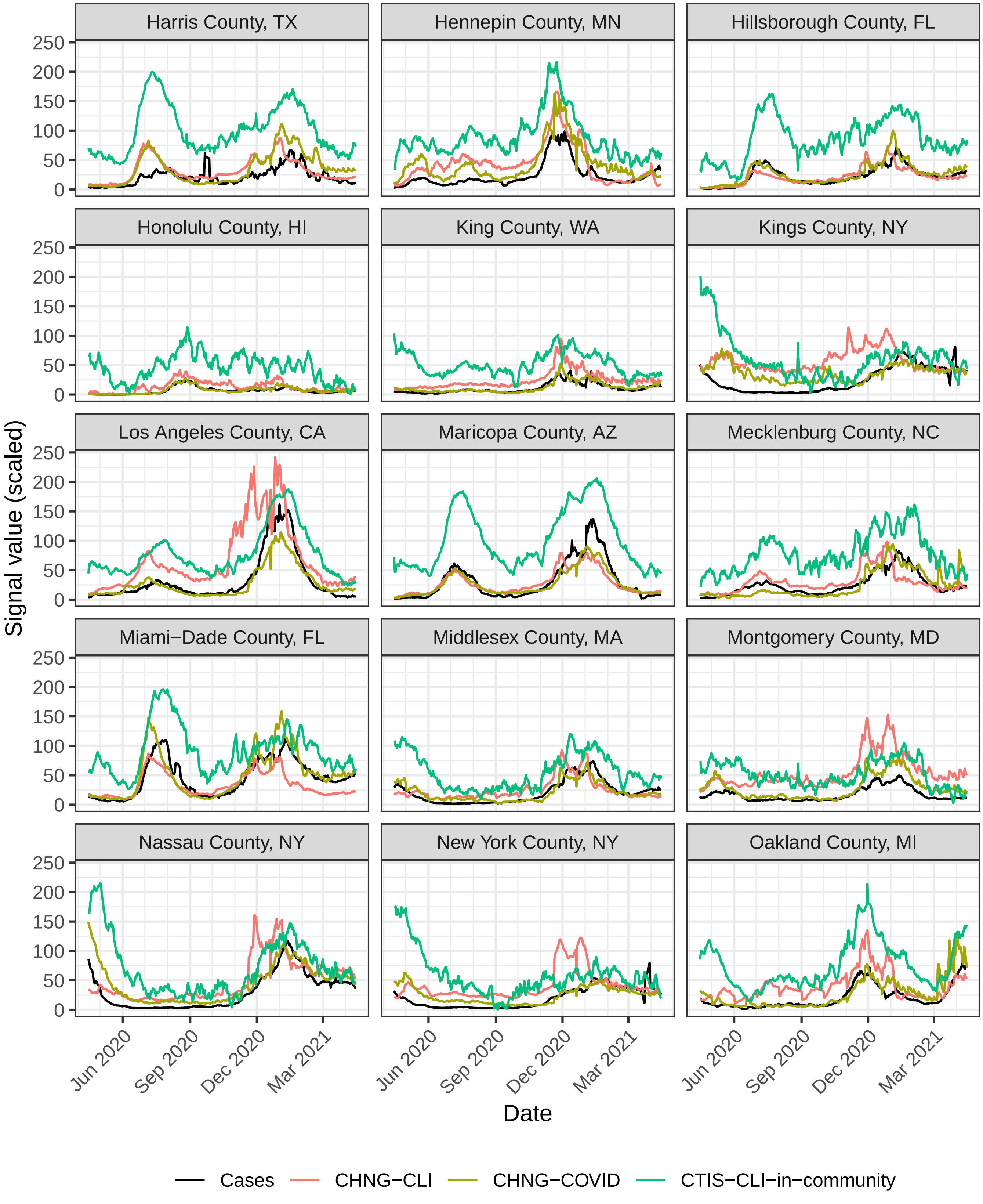

Fig. S8. Trends of cases, CHNG-CLI, CHNG-COVID, and CTIS-CLI-in-community for the 50 most populous U.S. counties. (Part 2 of 4.)

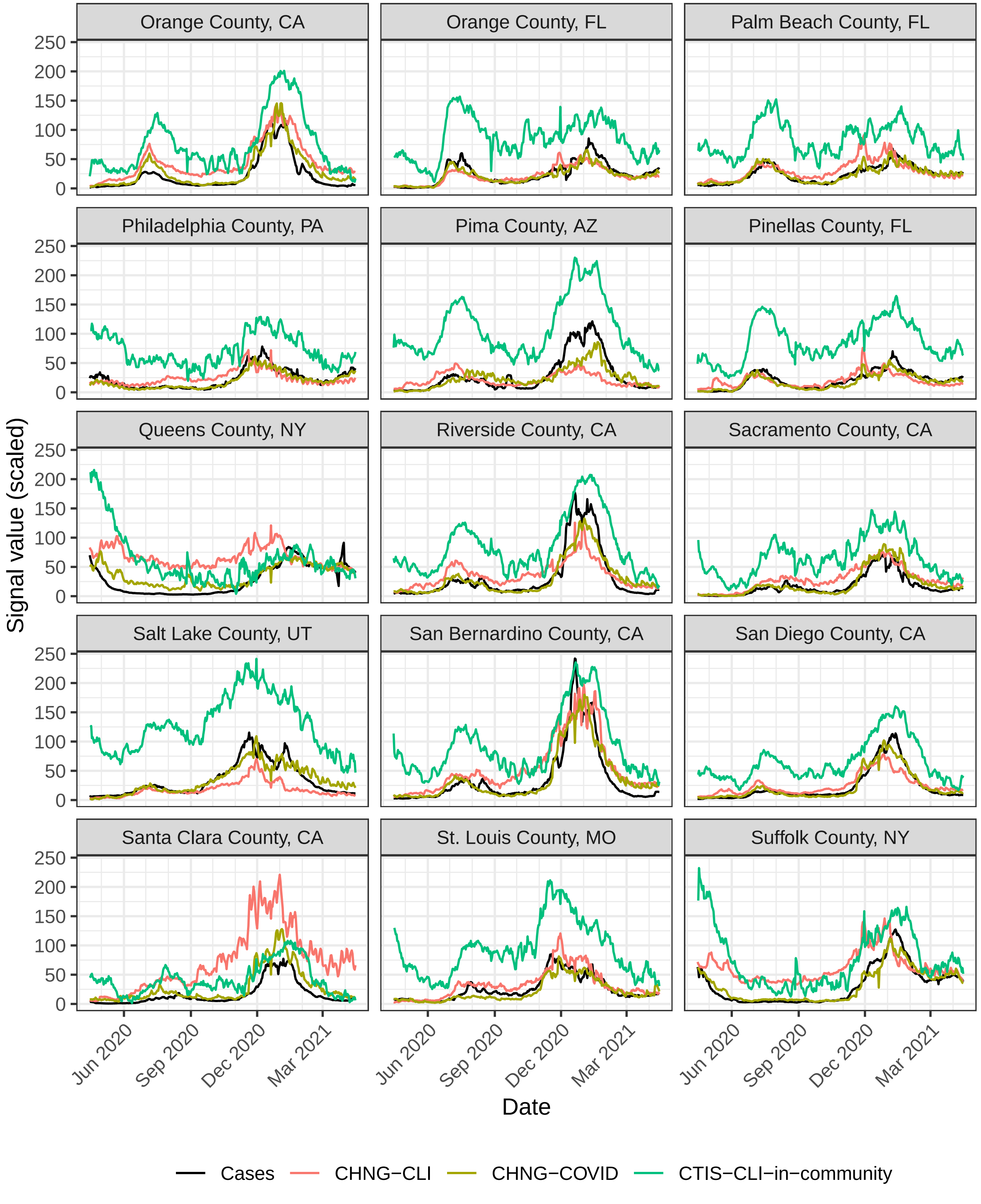

Fig. S9. Trends of cases, CHNG-CLI, CHNG-COVID, and CTIS-CLI-in-community for the 50 most populous U.S. counties. (Part 3 of 4.)

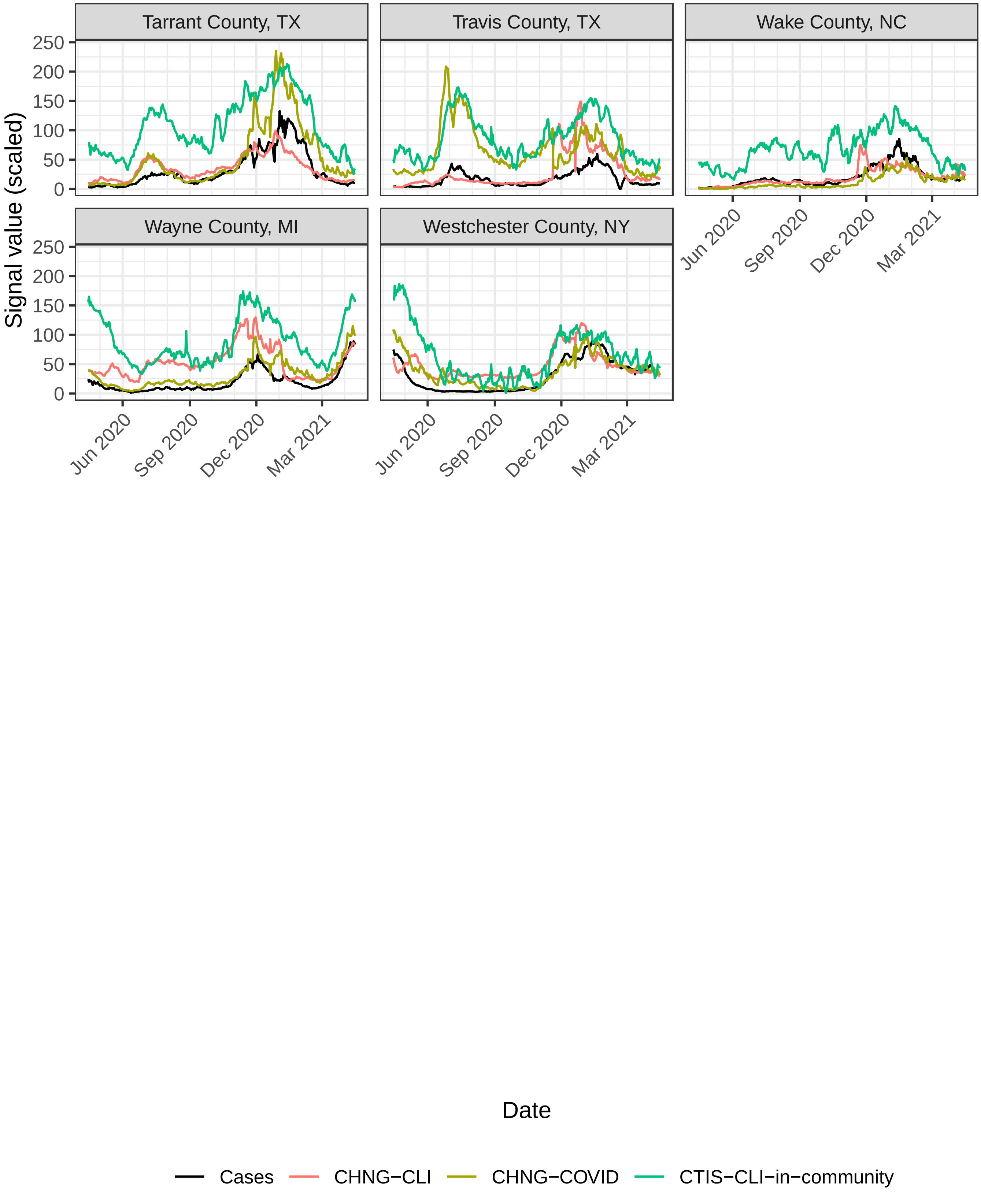

Fig. S10. Trends of cases, CHNG-CLI, CHNG-COVID, and CTIS-CLI-in-community for the 50 most populous U.S. counties. (Part 4 of 4.)

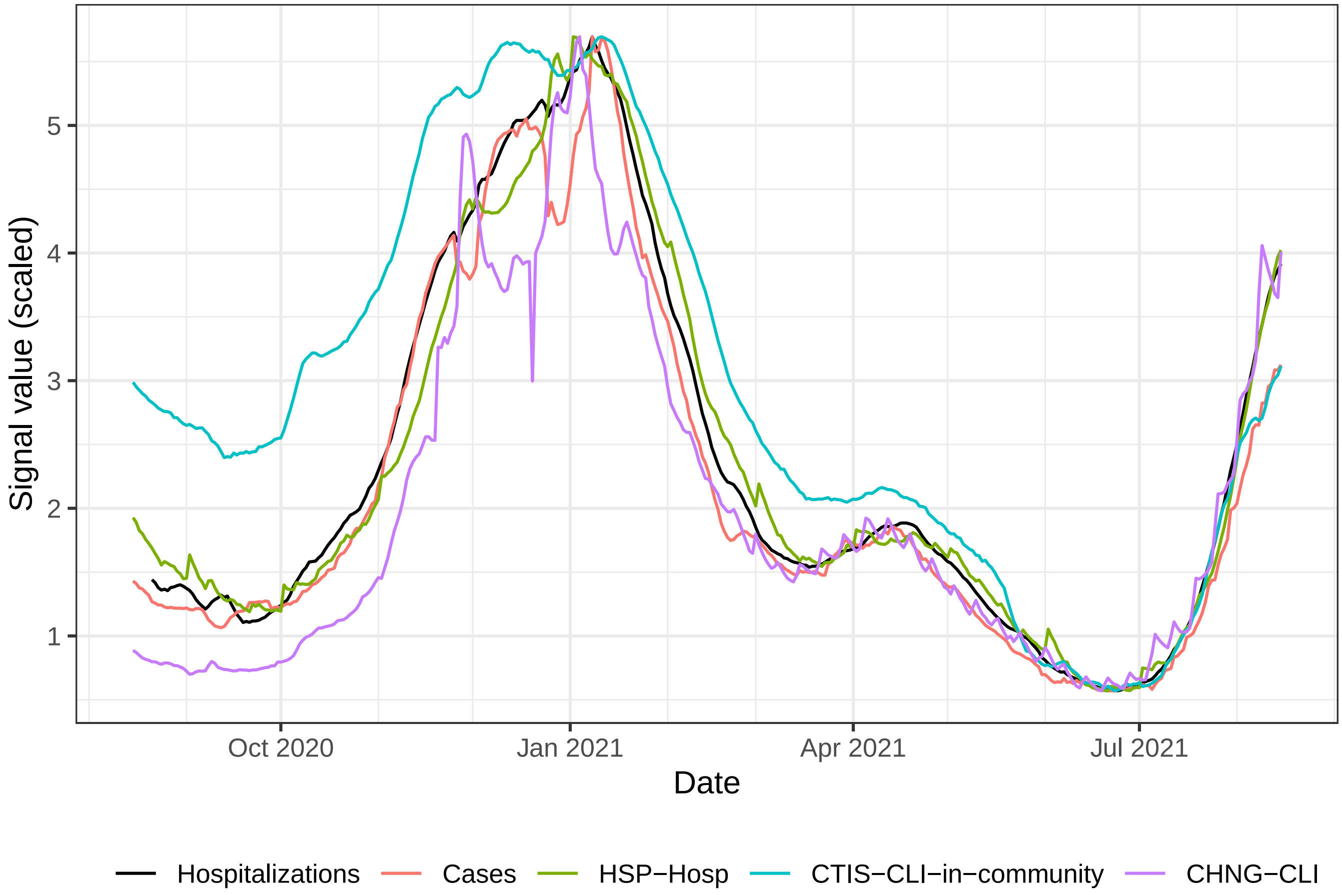

Fig. S11. National trends, from August 2020 to August 2021, of HHS-reported confirmed COVID-19 hospital admissions, along with several signals from the COVIDcast API. (HHS data was not consistently reported before August 2020; furthermore, as with cases in the previous trend plots, the HHS data has been smoothed using a 7-day trailing average.) Hospitalizations are displayed on the rate scale: counts per 100,000 people. Other signals are scaled to have the same range. HSP-Hosp is the percentage of new hospital admissions with COVID-associated diagnoses, based on claims data from health system partners (smoothed in time and adjusted for systematic day-of-week effects).

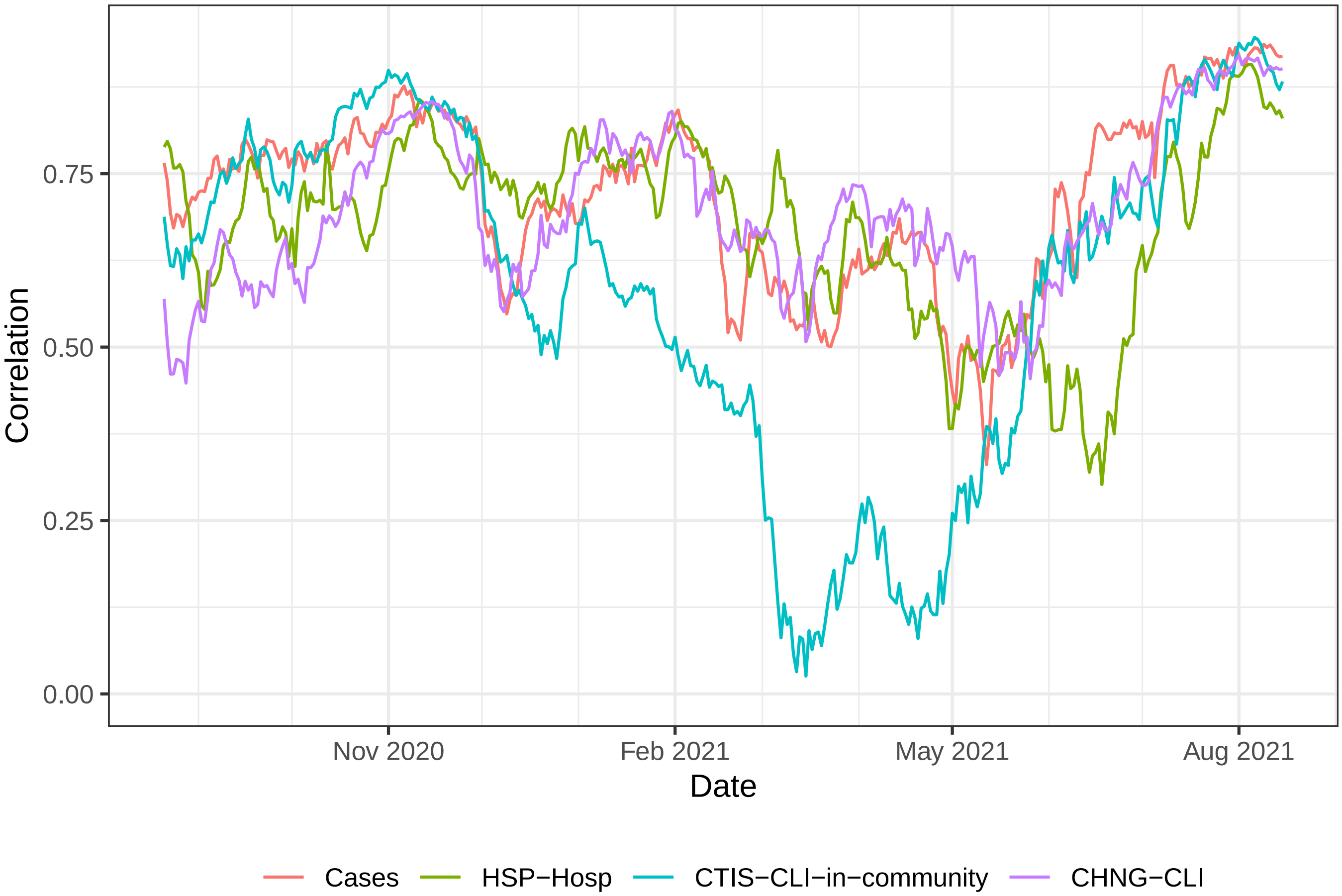

Fig. S12. Geo-wise correlations with hospitalization rates derived from HHS data, from August 15, 2020 to August 15, 2021, calculated for all times with sufficient available data within this period, over all state-like jurisdictions for which each signal was reported on at least 50 days during this period, limited to state-day combinations for which all signals are available.

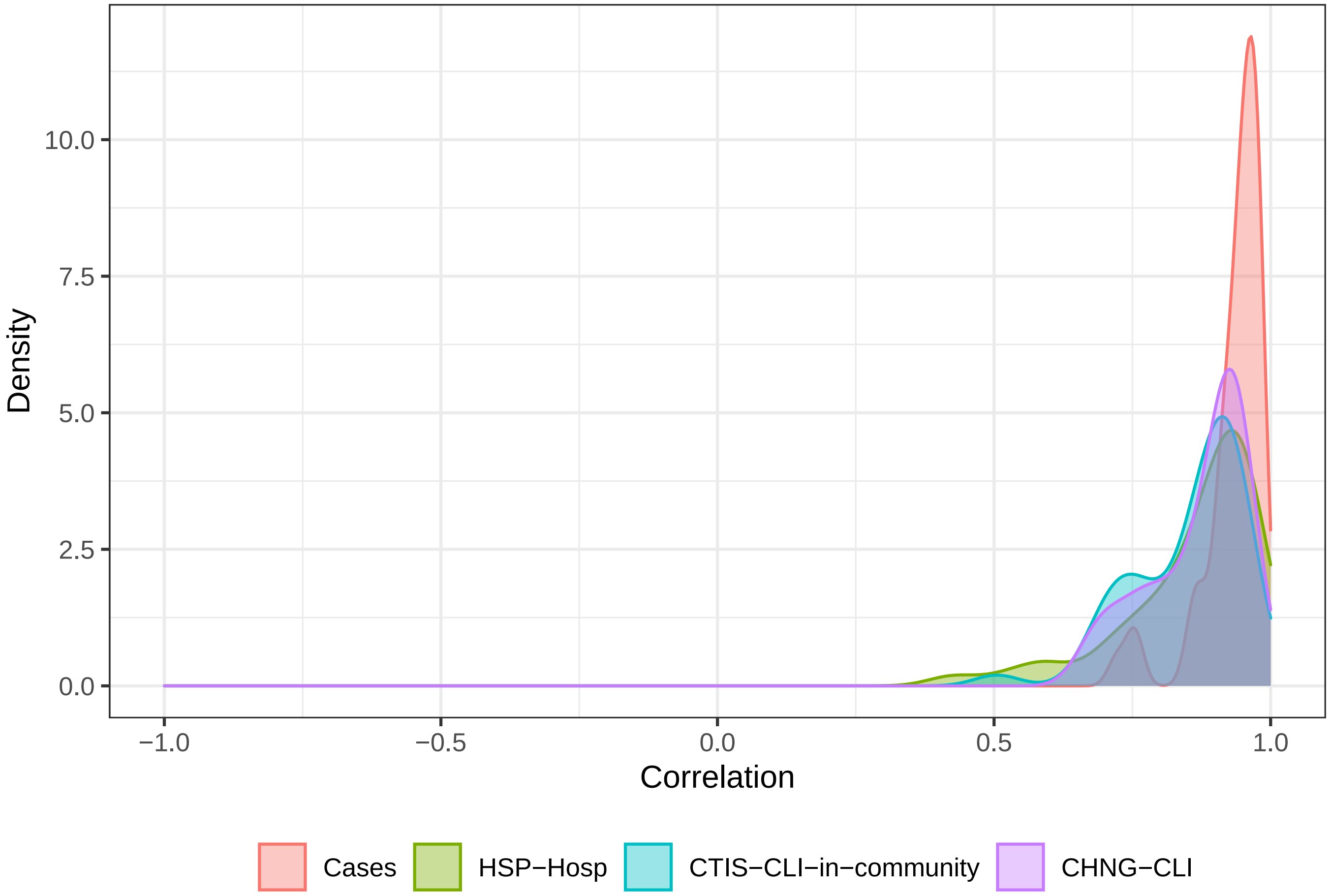

Fig. S13. Time-wise correlations with hospitalization rates derived from HHS data, from August 15, 2020 to August 15, 2021, calculated over all state-like jurisdictions for which each signal was reported on at least 50 days during this period, limited to state-day combinations for which all signals are available.
